# 

**Published:** 1866-01

**Authors:** 


### INDEX TO VOLUME XX.

A Bill to regulate the Practice of Dentistry 324
About the Editor of the Cosmos, etc. 89
Acknowledgement. Editorial 235
Address on Dental Science of To-day, by H. F. Bishop, D. D. S. 15
Address to Michigan Dental Association 126
Address, by George H. Cushing, D. D. S. 164
Address, by Dr. J. Chesebrough 171
A Description of Professor Vanderweyde's Apparatus for the Generation and Administration of Nitrous Oxide, and other Anæsthetics. 358
A File Carrier. (Editorial.) 284
A Funny Resolution 561
A Human Curiosity 322, 415
Alevolar Abscess, by Dr. A. Moore. 340
Aluminium as a Base for Artificial Teeth 410
American Dental Association 282, 416
Anæsthetics 40
Anæsthesia from the Internal use of Chloroform 80
An Address, by Dr. Geo. L. Paine 25
An Address, by Dr. Field 118
An Address, by Dr. I. J. Wetherbee, D. D. S. 380
A New Blow-pipe 45
A New Inhaler for all Anæsthetics 419
Another Death from Chloroform 321
Another Chloroform Case 413
A Prize Editorial. 373
A Simple, Safe and Easy Mode of preparing Oxygen 46
A Slight Eulogistic Mistake. (Editorial.) 190
A Useless Dispute Settled. (Editorial.) 517
Automatic Plugger. (Editorial.) 183
Bibliographical. (Editorial.) 54

Books. (Editorial.) 470
Bradley's Lip and Cheek Holder 284
Catarrhal Inflammation of the Antrum. 144
Central Ohio Dental Association 32, 53, 237
Central States Dental Association 237
Chloroform 322, 415
Chloroform and its Administration 377
Chloroform. Its Action and Administration 74
Chicago Dental Society 189
Close of the Volume 563
Code of Dental Ethics 343
Cold Application to Aching Teeth 63
Colorless Iodine. (Editorial.) 190
Commencement Exercises. (Editorial.) 145
Compensation 533
Constitution and By-Laws of the Central States Dental Association 301
Correspondence 204
Crystal Shred Gold 469
Diagnosis 66
Death from Chloroform 188
Dental Materia Medica 55, 101, 147, 193, 285, 424
Dental Education 242
Dental Education. (Editorial.) 469
Dental Education as Connected with Dental Associations 246
Dental Ethics 197
Dental Hobbies 205
Digestion 121
Died. (Editorial.) 192
Discussions of Central States Dental Association 259
Discussions of the Third Annual Meeting of the Central States Dental Association 540
Discussions of Mississippi Valley Dental Association 439
Display of Titles 510
Dissecting Abscesses of the Legs 85
Double Fracture of the Inferior Maxilla 38
Dr. Bogue's Improvement upon the Kingsley Palate 170
Effects of Tobacco on the Mental Powers 371
Eleventh Annual Meeting of Michigan Dental Association 133
Ether 371
Ether vs. Chloroform 412
Etherization 415
Examination for Degrees 99
Extracting Teeth 210, 528
External Periostitis 251
Exposed Pulps 407

Filling Teeth 331
Food and the Teeth 428
Foreign Correspondence 94
Gold Foil 542
Great Things 294
Ho! Every One to the Breeches. (Editorial.) 374
Hymenial. (Editorial.) 164
India Rubber Cones for Polishing Vulcanized Rubber 67
Indiana State Dental Association 340
Injection of Air into Abscesses and Fistulas 81
Instruction in Nitrous Oxide 470
Journalistic. (Editorial.) 140
Lady Hydrogen 519
Letter from Prof. C. W. Spalding 96
Little Things; by J. A. Robinson, D. D. S 218
Local Anæsthesia 362
Make the Foundation Firm 396
Marriage. (Editorial.) 192
Massachusetts Dental Society 315, 318
Measuring Cup. (Editorial.) 381
Medical Teachings. Selection 318
Medical Education—New Professorships in the Medical Department of Harvard University 318
Michigan Dental Association 48, 144
Minutes of the Twenty-second Annual Meeting of the Mississippi Valley Association of Dental Surgeons 307
Minutes of the Missouri State Dental Association 230
Minutes of the Ohio Dental Association 399
Miscellaneous Items 84
Mississippi Valley Dental Association 56, 238, 140
Monograph on Glycerine and Its Uses 86
More Light; by Dr A. Lawrence 220
Nashville Dental Society 236
Necrosis; by J. W. Bates, M. D 42
Necrosis of the Jaw from Decayed Tooth 46
New-York College of Dentistry 422
Nitrous Oxide Gas as an Anæsthetic 44, 467, 556
Nitrous Oxide Gas for Dental Anæsthesia 249
Nitrous Oxide Gas in Capital Operations 86
Nitrous Oxide Gas in Surgical Operations 85
Nitrous Oxide 88
Northern Ohio Dental Association 189
Obituary of Dr. W. K. Hodgen 47
Obituary. D. C. Ambler, M. D 328
Obituary. Dr. S. L. Hamlin 385

Obituary. Dr. Slack 376
Obituary. Dr. J. L. Nourse. 376
Obituaries 564
Ohio State Dental Society 502
Ohio Dental College Association. 143
On the Action of Medicinal Preparations of Iron on the Teeth 270
On Anæsthesia 363
On the Germinal Matter of the Blood, with Remarks upon the Formation of Fibrin 81
Operation for the Hare Lip, &c 272
Organization of the Ohio Dental Association. 372
Order of Business 48
Orthodontia 288
Phenic Acid as an Antiseptic 45
Personal. (Editorial.) 192
Personal and Selfish 119
Proceedings of the Central States Dental Association 252
Professional. (Editorial.). 191
Professional Character 333
Professional Liberty and Restraint 50
Professional Longings. 471
Pulp Extractor 420
Rhegoline—a Petroleum Naphtha for Producing Anæsthesia by Freezing 408
Removal of the Entire Tongue Successfully, during Life 371
Reproduction of Bone 84
Resolution of Mad River Valley Dental Association 100
Revenge. 389
Sixth Year Molars 161
Splitting Forceps. 124
Spontaneous Pulp Death 543
Stomatoscope 416
Stuffing Teeth 141, 299, 391
St. Louis Odontological Society. 322
Sulphate of Bebeeria in Facial Neuralgia. 273
Synopsis of Discussions of Missouri Dental Society 223
Ten Years in the Profession 495
The Agassiz Expedition 274
The American Dental Association 282
The Call. (Editorial.) 280
The Connecticut Valley Dental Association 544
The Duty of the Dentist to His Profession, His Patient, and to Himself 105
The Hypophosphites—Their Therapeutic Value 71
The New Year. (Editorial.). 49

The Ohio Dental College Association 52
The Ohio Dentists and the Goodyear Dental Vulcanite Company 518
The Province and Duties of the Dentist 19
Therapeutic Effects of Mercury in Acute Periostites 58
The Rattlesnake Poison 83
The Rubber Company and the American Dental Association 501
The Rubber Question. (Editorial.) 513
The Twenty-first Volume 564
The Use of the Mallet 483
Thoroughness in Dental Operations 168
Times. (Editorial.) 98
Use and Abuse of Poultices 369
Valedictory Address, by Prof. C. W. Spalding 153
Valedictory Address, by H. J. McKellops, D. D. S 536
Vital Function in Dentine 477
Wanderings of Pus 129
Western New York Dental Association 190
What we Most Need 297
White's Universal Port Polisher 53
Why Not? (Editorial.) 421

AMERICAN DENTAL ASSOCIATION—Meets annually (this year a Boston,) *Pres.* C. W. SPALDING, cf St. Louis, *Rec. Sec.* J. TAFT, of Cincinnati, *Cor. Sec.* GEO. W. ELLIS, of Philadelphia.

*List of Societies represented in the American Dental Association.*

ALBANY DENTAL ASSOCIATION—Meets monthly at Albany. *Pres.* ROBERT NELSON *Rec Sec.* W. F. WINNE, *Cor. Sec.* B. WOOD, of Albany.

BUFFALO DENTAL SOCIETY—Meets monthly at Buffalo. *Pres.* GEO. E. HAYES, *Rec Sec.* GEO. B. SNOW, of Buffalo.

BROOKLYN DENTAL ASSOCIATION—Meets semi-monthly *Pres.* W. C. PARKS, *Rec. Sec.* W. B. HURD, *Cor. Sec.* W. H. ATKINSON, of New York.

CINCINNATI DENTAL SOCIETY—Meets semi-monthly at Cincinnati. *Pres.* C. H. JAMES, *Rec. Sec.* WM. TAFT.

CENTRAL OHIO DENTAL ASSOCIATION—Meets Semi-annually* on the second Tuesday of May and November. *Pres.* Dr. J. ARMSTRONG, Bucyrus, O., *Sec.* A. W. MAXWELL Gallion, O.

CENTRAL STATES DENTAL ASSOCIATION—Meets annually* *Pres.* Dr. W H. MORGAN, *Sec.* W. H. SHADOAN, Louisville, Ky.

CENTRAL NEW YORK DENTAL ASSOCIATION Meets semi-annually. *Pres.* S. B. PALMER, *Rec. Sec.* S. G. MARTIN, *Cor. Sec.* P. HARRIS of Skaneatles.

CHICAGO DENTAL SOCIETY—Meets monthly at Chicago. *Pres.* E. W. HADLEY, *Rec. and Cor. Sec.* E. W. SAWYER of Chicago.

CONNECTICUT VALLEY DENTAL ASSOCIATION—Meets semi-annually, second Tuesday of May and October. *Pres.* F. SEARLE, *Rec. Sec* L. D. SHEPHERD, of Amherst, Mass.

HUDSON VALLEY DENTAL ASSOCIATION—meets monthly at Troy, N. Y. *Pres.* H. H. YOUNG, *Rec. Sec.* S. J. ANDRES, *Cor. Sec.* S. P. WELCH, of Troy.

INDIANA STATE DENTAL SOCIETY—Meets annually at Indianapolis on the last Tuesday of June. *Pres.* A. M. MOORE, *Rec. and Cor. Sec* JOS. RICHARDSON, of Terre Haute, Ind

IOWA STATE DENTAL SOCIETY—Meets semi-annually Jan. and July. *Pres.* L. C. INGERSOLL. *Rec. Sec.* H. S. CHASE.

MICHIGAN DENTAL SOCIETY—Meets annually fourth Tuesday of Jan. at Detroit. *Pres.* J. A. WATLING, *Rec. and Cor. Sec.* G. W. STONE, Albion.

MISSISSIPPI VALLEY DENTAL ASSOCIATION—Meets annually in February at Cincinnati, *Pres.* J. CHESEBROUGH. *Rec. Sec.* WM. TAFT, *Cor Sec.* H. A. SMITH, of Cin..

MISSOURI DENTAL ASSOCIATION—Meets on the first Tuesday of June, 1866, in St. Louis. *Pres.* Dr. H B McKELLOPS, St. Louis, *Sec* H. JUDD, St. Louis.

MERRIMAC VALLEY DENTAL SOCIETY—Meets semi annually* first Tuesday of Oct. and May. *Pres.* A. LAWRENCE, *Rec. Sec.* G. H. GERRY, of Lowell.

NEW YORK SOCIETY OF DENTAL SURGEONS—Meets semi-monthly in New York, *Pres.* W. N. ALLEN, *Rec. Sec.* C. D. ALLEN, *Cor. Sec.* C P. FITCH, of New York.

NEW HAVEN DENTAL SOCIETY—Meets on the first Wednesday of each month at New Haven. *Pres.* J. T. METCALF, *Rec and Cor. Sec.* C. L. SMITH, of New Haven.

NEW LONDON COUNTY DENTAL SOCIETY—Meets monthly.* *Pres.* R. W. BROWNE *Rec. Sec.* WM. ALLENDER.

NORTHERN OHIO DENTAL ASSOCIATION—Meets Semi-annually, (first Tuesday of May and October ) in Cleveland. *Pres.* B. STRICKLAND, *Rec. Sec.* L. BUFFETT, *Cor. Sec* B. F. ROBINSON.

OHIO DENTAL COLLEGE ASSOCIATION—Meets annually in February at Cincinnati. *Pres.* G. H. CUSHING, *Rec. Sec.* J TAFT, of Cincinnati.

ODONTOGRAPHIC SOCIETY OF PENNSYLVANIA—Meets monthly in Philadelphia. *Pres.* C. A. KINGSBURY, *Rec. Sec.* R. J. HORNER, *Cor. Sec.* J. H. McQUILLEN, of Phila.

PENNSYLVANIA ASSOCIATION OF DENTAL SURGEONS—Meets monthly in Phila. *Pres.* W W. FOUCHE, *Rec. Sec* JAMES TRUMAN *Cor. Sec.* G. W. ELLIS, of Philadelphia.

PITTSBURG DENTAL ASSOCIATION Meets monthly, (second Tuesday,) in Pittsburg. *Pres.* M. E. GILLESPIE, *Rec. Sec.* J. D WHITE.

PENINSULAR DENTAL SOCIETY—Meets quarterly.* *Pres.* SAMUEL MARSHALL, *Rec. Sec.* W. G. A. BONWILL, *Cor. Sec.* S. S. NONES.

WESTERN DENTAL SOCIETY—Meets annually.* *Pres.* T. P. ABELL, *Rec. Sec.* C. W. SPALDING, *Cor. Sec.* E. A BOGUE.

ST. LOUIS DENTAL SOCIETY—Meets monthly at St. Louis.

AMERICAN DENTAL CONVENTION—Meets annually first Tuesday of August, (next meeting at New York,) *Pres.* H. E. PEEBLES, *Rec. Sec.* G. H. A. SMITH. *Cor. Sec.* W. H. ALLEN, of New York.

**Place of meeting fixed by appointment.*

### CONTRIBUTORS.

W. H. ATKINSON, M. D., D. D. S.

B. WOOD, M. D.

W. H. SHADOAN.

WM. A. PEASE, D. D. S.

LEON F. HARVEY, M. D.

H. A. SMITH D. D. S.

GEO. B. SNOW, D. D. S.

WM. O. KULP.

A. S. TALBERT, D. D. S.

JOS. RICHARDSON, D. D. S.

A. LAWRENCE.

H. S. CHASE, D. D. S.

R. J. POORE.

GEO. L. PAINE, D. D. S.

A. A. BLOUNT, M. D.

J. S. LATIMER, D. D. S.

J. D. ANDERSON, D. D. S.

H. BENEDICT, D D. S.

J. MANSFIELD, D. D. S.

J. F. JOHNSON, D. D. S.

A. M. LESLIE, D. D. S.

GEO. WATT, D. D. S.

J. TAFT, D. D S.

S. B. PALMER.

F. ABBOTT.

T B. WELCH.

A. BERRY, D. D S.

A. W. MAXWELL.

H. F. BISHOP.

### EXCHANGES,

ST. LOUIS MEDICAL AND SURGICAL JOURNAL,
BOSTON MEDICAL AND SURGICAL JOURNAL,
THE PACIFIC MEDICAL AND SURGICAL JOURNAL,
BUFFALO MEDICAL AND SURGICAL JOURNAL,
OHIO MEDICAL AND SURGICAL JOURNAL,
PHILADELPHIA MEDICAL AND SURGICAL JOURNAL,
WESTERN LANCET AND OBSERVER,
CHICAGO MEDICAL JOURNAL,
DENTAL COSMOS,
L' ART DENTAIRE,
THE LONDON DENTAL REVIEW,
BRAITHWAITE'S RETROSPECT,
SAN FRANCISCO MEDICAL PRESS,
THE DENTAL TIMES,
THE PEOPLE' DENTAL JOURNAL,
LADIES' REPOSITORY,
HALL'S JOURNAL OF HEALTH,
DEUTSCHE VIERTELIAHRSSCHRIFT,
DENTAL CIRCULAR AND EXAMINER

### ORDER OF BUSINESS,

### Of the Twenty-Second Annual Meeting of the Mississippi Valley Dental Association.

1st. Report of officers.

2d. Election of officers, 10 A. M. 22d.

3d. Miscellaneous business. Reports of Committees in order at any interval in discussions.

4th. Essays or papers to be read on the opening of discussions.

SUBJECTS FOR DISCUSSION:

1st. Dental Materia Medica.

2d. Diseases of the teeth; Dental Physiology and Pathology.

3d. Filling teeth; methods and materials used.

4th. Dental Specialties.

5th. Mechanical Dentistry; New Inventions.

6th. Dental Education; Relation of Preceptor and Student.

G. W. KEELY,

J. P. ULREY,

J. G. VAN MARTER. } *Committee.*

---

### MICHIGAN DENTAL ASSOCIATION.

Albion, Jan. 1, 1866.

The eleventh annual meeting of the Michigan Dental Association, will be held at the Biddle House, in the city of Detroit, on Tuesday, January 30th, 1866, at 12 o'clock, noon, and will last three days.

It is earnestly hoped that the profession generally will turn out and give their hearty support to the advancement of dental science.

At the last regular meeting of the Association, the following resolution was adopted:

*Resolved*, That it is the duty of every member of this Association to furnish, at the next regular meeting, an essay, on some subject connected with the profession, that shall be submitted to the Committee on Publication, to be circulated among the members of this Association.

You are requested to invite your friends, who are regular Dentists, to be present on this occasion.

Respectfully, G. W. STONE, *Sec'y.*

### MISSISSIPPI VALLEY DENTAL ASSOCIATION.

The twenty-second annual meeting of the Mississippi Valley Dental Association, will begin on Wednesday, the 21st of February next, at 10 o'clock, A. M., in the lecture room of the Ohio Dental College. There will doubtless be a large gathering of the profession at that time. There are still some of the older members remaining, who feel an interest in and attachment to this society, that they can scarcely ever expect to entertain for any other. It is the oldest dental society in existence. A few of the present members have been with it from the begining. The early associations thus formed, have left impressions that will never be erased; to them the meetings of this society come speaking of the vicissitudes and trials through which it has passed; they also bring back the memory of those much beloved brethren, who have in one way or other passed away from our midst, and are no longer with us.

We hope to meet all the members at this meeting, and all others who can make it convenient to attend.

The order of business proposed by the executive committee. will be found on another page of this number.

### THE OHIO DENTAL COLLEGE ASSOCIATION.

This body will meet on Tuesday the 20th of February next, at 10 o'clock, A. M. at the lecture room of the college. It is very desirable that all the stockholders of the institution be present, as interests vital to the institution, will come up for consideration and action.

By virtue of the amendment to the charter, which was passed by the legislature last spring, it will devolve upon that meeting to elect a board of trustees, who shall have control of the interests of the college.

In the selection of this board, great care and wisdom should be exercised, for upon their action, to a large extent, depends its efficiency and usefulness.

These trustees will be chosen from the stock-holders, as it is to be presumed that they will feel more interest in the institution than those outside of the Association. Let every stock-holder be present, so far as it may be possible.

The college now seems to be entering upon a career of greater prosperity and usefulness than ever before. Let such measures be adopted, and means employed as shall secure still greater usefulness.

### BIBLIOGRAPHICAL.

AMERICAN DENTAL ASSOCIATION—Meets annually (this year a Boston,) *Pres.* C. W. SPALDING, cf St. Louis, *Rec. Sec.* J. TAFT, of Cincinnati, *Cor. Sec.* GEO. W. ELLIS, of Philadelphia.

*List of Societies represented in the American Dental Association.*

ALBANY DENTAL ASSOCIATION—Meets monthly at Albany. *Pres.* ROBERT NELSON *Rec Sec.* W. F. WINNE, *Cor. Sec.* B. WOOD, of Albany.

BUFFALO DENTAL SOCIETY—Meets monthly at Buffalo. *Pres.* GEO. E. HAYES, *Rec Sec.* GEO. B. SNOW, of Buffalo.

BROOKLYN DENTAL ASSOCIATION—Meets semi-monthly *Pres.* W. C. PARKS, *Rec. Sec.* W. B. HURD, *Cor. Sec.* W. H. ATKINSON, of New York.

CINCINNATI DENTAL SOCIETY—Meets semi-monthly at Cincinnati. *Pres.* C. H. JAMES, *Rec. Sec.* WM. TAFT.

CENTRAL OHIO DENTAL ASSOCIATION—Meets Semi-annually* on the second Tuesday of May and November. *Pres.* Dr. J. ARMSTRONG, Bucyrus, O., *Sec.* A. W. MAXWELL Gallion, O.

CENTRAL STATES DENTAL ASSOCIATION—Meets annually* *Pres.* Dr. W H MORGAN, *Sec.* W. H. SHADOAN, Louisville, Ky.

CENTRAL NEW YORK DENTAL ASSOCIATION Meets semi-annually. *Pres.* S. B. PALMER, *Rec. Sec.* S. G. MARTIN, *Cor. Sec.* P. HARRIS of Skaneatles

CHICAGO DENTAL SOCIETY—Meets monthly at Chicago. *Pres.* E. W. HADLEY, *Rec. and Cor. Sec.* E. W. SAWYER of Chicago.

CONNECTICUT VALLEY DENTAL ASSOCIATION—Meets semi-annually, second Tuesday of May and October. *Pres.* F. SEARLE, *Rec. Sec* L. D. SHEPHERD, of Amherst, Mass.

HUDSON VALLEY DENTAL ASSOCIATION—meets monthly at Troy, N. Y. *Pres.* H. H. YOUNG, *Rec. Sec.* S. J. ANDRES, *Cor. Sec.* S. P. WELCH, of Troy.

INDIANA STATE DENTAL SOCIETY—Meets annually at Indianapolis on the last Tuesday of June. *Pres.* A. M. MOORE, *Rec. and Cor. Sec* JOS. RICHARDSON, of Terre Haute, Ind

IOWA STATE DENTAL SOCIETY—Meets semi-annually Jan. and July. *Pres.* L. C. INGERSOLL, *Rec. Sec.* H. S. CHASE.

MICHIGAN DENTAL SOCIETY—Meets annually fourth Tuesday of Jan. at Detroit. *Pres.* J. A. WATLING, *Rec. and Cor. Sec.* G. W. STONE, Albion.

MISSISSIPPI VALLEY DENTAL ASSOCIATION—Meets annually in February at Cincinnati, *Pres.* J. CHESEBROUGH. *Rec. Sec.* WM. TAFT, *Cor Sec.* H. A. SMITH, of Cin..

MISSOURI DENTAL ASSOCIATION—Meets on the first Tuesday of June, 1866, in St. Louis. *Pres.* Dr. H. B. McKELLOPS. St. Louis, *Sec* H. JUDD, St. Louis.

MERRIMAC VALLEY DENTAL SOCIETY—Meets semi annually* first Tuesday of Oct. and May. *Pres.* A. LAWRENCE, *Rec. Sec.* G. H. GERRY, of Lowell.

NEW YORK SOCIETY OF DENTAL SURGEONS—Meets semi-monthly in New York, *Pres.* W. N. ALLEN, *Rec. Sec.* C. D. ALLEN, *Cor. Sec.* C. P. FITCH, of New York.

NEW HAVEN DENTAL SOCIETY—Meets on the first Wednesday of each month at New Haven. *Pres.* J. T. METCALF, *Rec. and Cor. Sec.* C. L. SMITH, of New Haven.

NEW LONDON COUNTY DENTAL SOCIETY—Meets monthly.* *Pres.* R. W. BROWNE *Rec. Sec.* WM. ALLENDER.

NORTHERN OHIO DENTAL ASSOCIATION—Meets Semi-annually, (first Tuesday of May and October.) in Cleveland. *Pres.* B. STRICKLAND, *Rec. Sec.* L. BUFFETT, *Cor. Sec* B. F. ROBINSON.

OHIO DENTAL COLLEGE ASSOCIATION—Meets annually in February at Cincinnati. *Pres.* G. H. CUSHING, *Rec. Sec.* J. TAFT, of Cincinnati.

ODONTOGRAPHIC SOCIETY OF PENNSYLVANIA—Meets monthly in Philadelphia. *Pres.* C. A. KINGSBURY, *Rec. Sec.* R. J. HORNER, *Cor. Sec.* J. H. McQUILLEN, of Phila.

PENNSYLVANIA ASSOCIATION OF DENTAL SURGEONS—Meets monthly in Phila. *Pres.* W. W. FOUCHE, *Rec. Sec* JAMES TRUMAN *Cor. Sec.* W. ELLIS, of Philadelphia.

PITTSBURG DENTAL ASSOCIATION—Meets monthly, (second Tuesday,) in Pittsburg *Pres.* M. E. GILLESPIE, *Rec. Sec.* J. D. WHITE.

PENINSULAR DENTAL SOCIETY—Meets quarterly.* *Pres.* SAMUEL MARSHALL, *Rec. Sec.* W. G. A. BONWILL, *Cor. Sec.* S. S. NONES.

WESTERN DENTAL SOCIETY—Meets annually.* *Pres.* T. F. ABELL, *Rec. Sec.* C. W SPALDING, *Cor. Sec.* E. A BOGUE.

ST. LOUIS DENTAL SOCIETY—Meets monthly at St. Louis.

AMERICAN DENTAL CONVENTION—Meets annually first Tuesday of August, (nex meeting at New York,) *Pres.* H. E. PEEBLES, *Rec. Sec.* J. H. A. SMITH. *Cor. Sec.* W. H. ALLEN, of New York.

**Place of meeting fixed by appointment.*

### CONTRIBUTORS.

W. H. ATKINSON, M. D., D. D. S.

B. WOOD, M. D.

W. H. SHADOAN.

WM. A. PEASE, D. D. S.

LEON F. HARVEY, M. D.

H. A. SMITH. D. D. S.

GEO. B. SNOW, D. D. S.

WM. O. KULP.

A. S. TALBERT, D. D. S.

JOS. RICHARDSON, D. D. S.

A. LAWRENCE.

H. S. CHASE, D. D. S.

R. J. POORE.

GEO. L. PAINE, D. D. S.

A. A. BLOUNT, M. D.

G. A. MILLS.

C. W. SPALDING, D.D.S.

J. S. LATIMER, D. D. S.

J. D. ANDERSON, D. D. S.

H. BENEDICT, D D. S.

J. MANSFIELD, D. D. S.

J. F. JOHNSON, D. D. S.

A. M. LESLIE, D. D. S.

GEO. WATT, D. D. S.

J. TAFT, D. D. S.

S. B. PALMER.

F. ABBOTT.

A. BERRY, D. D S.

A. W. MAXWELL.

H. F. BISHOP.

JOSEPH S. HILDRETH, M..D

GAM'L JACKSON.

H. T. CROSSINGER.

### EXCHANGES,

ST. LOUIS MEDICAL AND SURGICAL JOURNAL,
BOSTON MEDICAL AND SURGICAL JOURNAL,
THE PACIFIC MEDICAL AND SURGICAL JOURNAL,
BUFFALO MEDICAL AND SURGICAL JOURNAL,
OHIO MEDICAL AND SURGICAL JOURNAL,
PHILADELPHIA MEDICAL AND SURGICAL JOURNAL,
WESTERN LANCET AND OBSERVER,
CHICAGO MEDICAL JOURNAL,
DENTAL COSMOS,
L' ART DENTAIRE,
THE LONDON DENTAL REVIEW,
BRAITHWAITE'S RETROSPECT,
SAN FRANCISCO MEDICAL PRESS,
THE DENTAL TIMES,
THE PEOPLE'S DENTAL JOURNAL,
LADIES' REPOSITORY,
HALL'S JOURNAL OF HEALTH,
DEUTSCHE VIERTELIAHRSSCHRIFT,
DENTAL CIRCULAR AND EXAMINER
CHICAGO MEDICAL EXAMINER.
THE CINCINNATI JOURNAL OF MEDICINE.

### CONTENTS.

ORIGINAL ESSAYS AND COMMUNICATIONS.

Dental Materia Medica ........ 9

Address ........ 14

The Province and Duties of the Dentist ........ 19

Address ........ 25

PROCEEDINGS OF SOCIETIES.

Central Ohio Dental Association ........ 32

SELECTIONS.

Double Fracture of the Inferior Maxilla ........ 38

Anæsthetics ........ 40

The Preparation of Pure Chloroform ........ 41

Necrosis ........ 42

Nitrous Oxide as an Anæsthetic ........ 44

Phenic Acid as an Antiseptic ........ 45

A New Blow-Pipe ........ 45

A Simple, Safe and Easy Mode of Preparing Oxygen ........ 46

Necrosis of the Jaw from Decayed Tooth ........ 46

Obituary of Dr. W. R. Hodgen ........ 47

Order of Business ........ 48

Michigan Dental Association ........ 48

EDITORIAL.

The New Year ........ 49

Professional Liberty and Restraint ........ 50

Central Ohio Dental Association ........ 52

Mississippi Valley Dental Association ........ 53

White's "Universal Porte Polisher." ........ 53

The Ohio Dental College Association ........ 53

Bibliographical ........ 54

---

### The Dental Register

### OF THE WEST.

*Issued Monthly, at $3 a Year in Advance.*

DEVOTED TO THE INTERESTS OF THE DENTAL PROFESSION.

**EDITED BY J. TAFT**

The volume commences in January and closes in December.

The Register being issued monthly is always fresh, therefore indispensable to the practitioner who desires to keep posted up with the new inventions and improvements which are daily developed. Neither expense nor effort will be spared to give value and variety to its contents. Articles will be illustrated with well executed engravings whenever they will add to the interest or assist in explanation.

Its extensive circulation and frequency of issue makes it one of the BEST DENTAL ADVERTISING MEDIUMS in the United States.

RATES OF ADVERTISING.

One page, 1 year, $50. Half page, 1 year, $30. Quarter page, or card, 1 year $20.

All advertising bills payable in advance, or at furthest after the issue of the first number containing the advertisement. All transient advertisements to be paid for in advance.

☞ CONTRIBUTIONS SOLICITED.

Address, J. TAFT, or "DENTAL REGISTER," Cincinnati, Ohio.

Business Communications to

**J. TAFT, Publisher,**
No. 172 Race Street.

